# Post-operative septic arthritis after arthroscopy: modern diagnostic and therapeutic concepts

**DOI:** 10.1007/s00167-021-06525-8

**Published:** 2021-03-23

**Authors:** Andreas Voss, Christian G. Pfeifer, Maximilian Kerschbaum, Markus Rupp, Peter Angele, Volker Alt

**Affiliations:** 1 Department of Trauma Surgery, University Medical Center Regensburg, Franz-Josef-Strauss-Allee 11, 93053 Regensburg, Germany; 2 Sporthopaedicum, Regensburg, Straubing, Germany

**Keywords:** Joint infection, Complication, Arthroscopy, Antibiotics, Shoulder, Knee, Ankle, Hip, Wrist, Elbow

## Abstract

**Purpose:**

Septic arthritis is a significant complication following arthroscopic surgery, with an estimated overall incidence of less than 1%. Despite the low incidence, an appropriate diagnostic and therapeutic pathway is required to avoid serious long-term consequences, eradicate the infection, and ensure good treatment outcomes. The aim of this current review article is to summarize evidence-based literature regarding diagnostic and therapeutic options of post-operative septic arthritis after arthroscopy.

**Methods:**

Through a literature review, up-to-date treatment algorithms and therapies have been identified. Additionally, a supportive new algorithm is proposed for diagnosis and treatment of suspected septic arthritis following arthroscopic intervention.

**Results:**

A major challenge in diagnostics is the differentiation of the post-operative status between a non-infected hyperinflammatory joint versus septic arthritis, due to clinical symptoms, (e.g., rubor, calor, or tumor) can appear identical. Therefore, joint puncture for microbiological evaluation, especially for fast leukocyte cell-count diagnostics, is advocated. A cell count of more than 20.000 leukocyte/µl with more than 70% of polymorphonuclear cells is the generally accepted threshold for septic arthritis.

**Conclusion:**

The therapy is based on arthroscopic or open surgical debridement for synovectomy and irrigation of the joint, in combination with an adequate antibiotic therapy for 6–12 weeks. Removal of indwelling hardware, such as interference screws for ACL repair or anchors for rotator cuff repair, is recommended in chronic cases.

**Level of evidence:**

IV.

## Introduction

In recent years, arthroscopic interventions have had a revolutionary impact on treating joint pathologies. Due to the technical improvements in arthroscopy, more and more pathologies are treated with a minimally invasive approach enabling the surgeon to have the best view during surgical intervention. As with every surgery, there is always the risk of infection, which has a major impact on the clinical outcome of every patient treated, through an arthroscopic technique. Even though it is considered to be a rare complication with an overall estimated incidence of less than 1%, the timely diagnosis and treatment of an infected joint is extremely important for successful management [7, 70]. However, an analysis of 15,167 patients after knee and shoulder arthroscopy showed that 37.1% of patients were readmitted within 30 days post-surgery due to an infection, underscoring the importance of post-surgical septic arthritis [82].

## Epidemiology and pathophysiology

### Shoulder

The infection can evolve via a hematogenous scattering or direct entry into the immune-privileged joint, which will be the focus of this article. Following shoulder arthroscopy with the use of anchors or suture material, germs can find excellent conditions for settlement.[22, 72]. Previous studies have identified significant risk factors for shoulder joint infections, which account for 88% of the patients examined in these studies (Table 1) [6, 13, 42].

**Table 1:** Risk factors of shoulder infection [4, 6, 18, 56] (list not exhaustive)

Alcohol abuse COPD Omarthrosis Tuberculosis Urinary catheter Diabetes mellitus Smoking Hyperuricemia Cirrhosis i.v. catheter Male sex	Drug abuse Systemic immunosuppression (medicinal, HIV) Systemic diseases (e.g., Hodgkin's lymphoma, chronic lymphocytic leukemia, rheumatoid arthritis, and gout) Renal failure Obesity Menstruation, pregnancy: increased risk of gonorrhea Malnutrition

Predominantly, shoulder infections are iatrogenic through peri- or intra-articular infiltration, as well as through surgical interventions [29]. There is an increased likelihood of shoulder joint infection in open procedures [24, 38] compared to purely arthroscopic procedures, that have an overall post-operative overall of around 1% [7, 84] and ranges from 0.16 to 2.10%, if it includes revision arthroscopic interventions [11, 54, 55, 58, 76, 84]. Almost all infections with a positive germ cultivation showed the presence of *Cutibacterium acnes* or *Staphylococci*. However, some other germs such as *Pseudomonas aeruginosa*, *Mycobacterium tuberculosis*, and *Actinomyces* have also been reported. There is an ongoing discussion about the use of pre-operative skin disinfectants and whether this has an influence on the post-operative infection rate. Therefore, Saltzmann et al. [61] investigated the use of povidone-iodine and showed an increased colonization in 31% of the cases following skin disinfection, 19% after iodophor-isopropyl alcohol disinfection and 7% after chlorhexidine-isopropyl alcohol disinfection. Additionally, a specific investigation regarding *Cutibacterium acnes* showed a persistence of colonization in 22.8% of the cases after pre-operative skin disinfection, with an increased colonization rate (42.6%) at the end of surgery [69]. In a time-related investigation, the odds ratio for a post-operative infection is 3.6 when surgery takes longer than 45 min with a more protective ratio for shorter interventions [11, 69]. To summarize the findings, the incidence of post-operative infection in shoulder arthroscopy is multifactorial and depends upon the type and time of surgery (primary or revision), and associated risk factors, and may also be influenced by the type of disinfectant,

### Elbow joint infections

Despite the shift from open to arthroscopic procedures, the real incidence of infection at the beginning was unknown. Following a comprehensive analysis of 2704 Medicare patients treated with elbow arthroscopy, an incidence rate of 1.5% for deep infections has been reported [17]. There are only limited data regarding risk factors for elbow infection after arthroscopy, compared to knee and shoulder. However, it has been shown that alcohol use, inflammatory arthritis, hypercoagulability, age (> 65 years), diabetes mellitus, intra-articular corticosteroid, and obesity are significant risk factors [17, 81].

### Wrist joint infections

Most of the data presented are referred to atraumatic septic arthritis with 2–5 infections per 100,000 in the general population and up to 38 per 100,000 individuals with rheumatoid arthritis [62]. The infection rate after arthroscopy can only be estimated from case series and is reported to be between 0 and 0.6% [8, 36, 67, 68, 83]. One study investigating the complications after wrist arthroscopy of 10,107 patients reported an incidence rate of 0.04% [45]. Furthermore, it has to be mentioned that the authors noted that infections were either not recorded or reported as infrequent [45].

### Hip joint infections

Due to multifactorial etiologies of hip pain, intra-articular anesthetic or cortisone injection, as well as the injection of agents for MRI (gadolinium-based contrast agents) or CT have become essential tools in diagnosing hip pathologies. Wang et al. showed that there is a correlation between post-operative infection and pre-operative infiltration [78]. The closer the infiltration is performed prior to the surgical procedure, then the risk of infection increases after hip arthroscopy. The overall infection rate after hip arthroscopy was 1.1% (86/7620) with an elevated infection rate after injection of up to 2.8% with an injection less than 3 months before surgery (rate of control group was 1.1%) [78].

Summarizing the reported incidence for infection after hip arthroscopy in the literature, the rate is between 0 and 1.2%.[16, 20, 23, 26, 32, 34, 47, 53, 57, 75, 78, 79]. Regarding the risk factors for an infection, the reported factors are similar to the risk factors reported for shoulder arthroscopy (Table 2).

**Table 2:** Independently associated factors with increased infections risk after hip arthroscopy [78]

Pre-operative joint injections Smoking Depression Hyperlipidemia Hypertension	Hemodialysis Obesity Inflammatory arthritis Coronary artery disease Hypothyroidism Chronic kidney disease

### Knee joint infections

The overall reported infection rate is between 0.1 and 1.8%.[5, 10, 14, 18, 19, 21, 33, 37, 50, 66, 77]. This includes recent but also older literature. The real rate might be lower, as reported by meta-analysis of Cancienne et al.[19] and Yeranosian et al. [84, 85], who independently investigated the infection rate of over 1,000,000 patients after knee arthroscopy. They identified a post-arthroscopy infection risk between 0.15 and 0.46%, depending upon the cohort and the type of procedure. Additionally, demographic variables and comorbidities such as age (< 65), male gender, morbid obesity (BMI 40 +), tobacco use, diabetes mellitus, inflammatory arthritis, congestive heart failure, chronic kidney disease, hemodialysis, hypercoagulable disorder, and depression have been identified as independent risk factors for an infection after knee arthroscopy [19]. Besides the reported incidence and risk factors, there is a lot of information about the germs related to knee infection and *Staphylococcus aureus* is by far the most found bacteria [3, 5, 70]. However, some other germs such as coagulase-negative staphylococci (e.g., *Staphylococcus epidermidis)*, MRSA, enterobacteria, streptococci, or fungal pathogens have been reported [3, 5, 70].

A significantly higher rate of post-surgical infections after arthroscopic ACL repair is described for professional athletes compared to amateur athletes [71]. However, papers by our group and others revealed no significant increase in infection rate after ACL reconstruction in professional athletes [12, 44].

Krutsch et al. described sports-related differences in infection rates after ACL injury and reconstruction [44]. Athletes practicing summer outdoor sports (e.g., football) had a significantly higher risk for infection after ACL reconstruction than winter sport athletes [44].

### Ankle joint infections

Comprehensive registry analysis are missing, although the overall rate is reported to be between 0.13 and 1.8% [1, 9, 28, 29, 80]. It is even higher in patients who received an intraoperative intra-articular corticosteroid injection with an incidence rate of 3.9% [80].

## Diagnosis

The difficulty in the diagnostic approach is to distinguish between a real post-operative septic arthritis and a post-operative hyperinflammation. The classical signs of infection such as joint swelling, reddening, overheating, pain, and limited range of motion (tumor, rubor, dolor, calor, and functio laesa) can be seen, whereas fever (possibly also chills) is more likely to be seen in septic arthritis. The diagnosis may not be obvious and mentioned signs of a joint infection can be masked [50]. Therefore, mild symptoms due to infection can be masked as signs of normal post-operative hyperinflammation [15, 40, 51]. According to Schollin-Borg et al.[64], in 60% of their cases after ACL reconstruction, the diagnosis of infection was missed at the patients’ first visit. Specifically, this is the case for patients with an indolent joint infection with non-aggressive or moderately aggressive germs, such as *coagulase-negative Staphylococcus,* and especially with *Cutibacterium acnes* after shoulder arthroscopy [50]. Additionally, gout arthritis should also be excluded, which can be done by the interpretation of blood infection parameters and examination results from joint fluid samples (joint fluid microscopy to confirm or exclude crystals).

After an inspection and palpation, the painful restricted range of motion can be the leading symptom during clinical examination [48]. Additionally, it is essential to distinguish between a joint irritation and a joint infection, especially after previous surgery (Table 3) [65].

**Table 3:** Criteria for differentiation between joint irritation and joint infection (modified according to [65], CRP = C-reactive protein)

Pro joint irritation	Pro joint infection
Symptoms < 12 h after intervention	Symptoms 12 h to 5 days after the intervention
Joint swelling	General feeling of sickness
No fever	Fever (but not mandatory)
Only a slight increase of CRP	Significant increase of CRP
Leukocytes < 20.000/µl	Leukocytes > 20.000/µl
Normal procalcitonin	Increased procalcitonin
No risk factor (see Table 1)	One or more risk factors

### Additional diagnostics

Even if there is little suspicion of an infected joint, blood tests should be initiated. Particular attention should be paid to the determination of the infection parameters such as leukocyte count, C-reactive protein (CRP), and the procalcitonin (PCT). Additionally, kidney and liver parameters should be determined, as they can be helpful to initiate and adapt a later antibiotic therapy. If there are signs of systemic infection, (e.g., fever), blood cultures should be taken at least in 2 pairs—2 aerobic and 2 anaerobic cultures from 2 different sites. However, the informative value of solely chemical blood tests, only shows a low specificity. The sensitivity can be increased by determining interleukin-6 in addition to CRP [65].

By ultrasound examination, a quick and easy-to-use procedure is available that allows for the detection of peri-articular fluid accumulation and joint effusions. However, it cannot distinguish between hyperinflammation and septic arthritis, as both show similar findings.

If infection is suspected, a conventional radiograph (2 plains) of the affected joint should also be carried out. This allows for assessment of any bony changes (e.g., osteolysis and osteophytes), as well as the evaluation of a physiological joint position and possible implants.

Extended diagnostic imaging with computed tomography (CT) or magnetic resonance imaging (MRI, with, i.v. injection of contrast medium) helps to further investigate the involvement of peri-articular soft-tissue structures and determination of an abscess. Positron emission tomography (PET)-CT and leukocyte scintigraphy are indicated to clarify unclear constellations of infection, especially in cases with unclear infection parameters, but are not used as a primary detection tool for joint infections.

The essential diagnostic tool for a suspected joint infection is the joint puncture. A sonographically assisted puncture is recommended and allows the controlled needle placement of the target area [2]. The procedure should be performed under sterile conditions (special room, disinfection, mouth protection, sterile gloves, and sterile drape). Afterward, the joint fluid sample should be assessed macroscopically (serous, clear, cloudy, and bloody) and then used for further determination of cell count, gram staining, microscopy, and extended microbiological diagnostics.

**Fig. 1:**
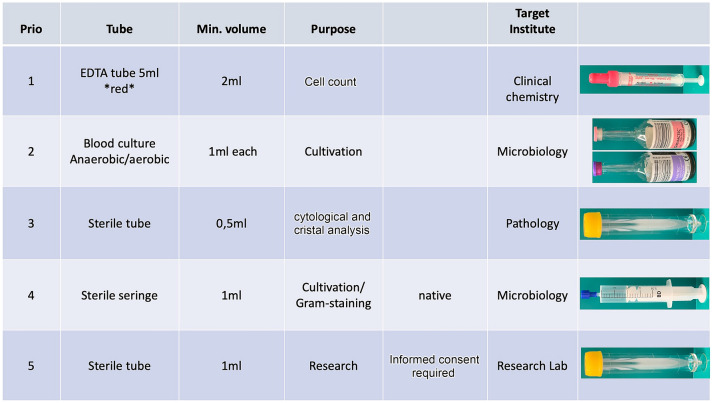
Priority protocol for suspected joint infections. Depending on the amount of joint fluid, the user should start with priority 1 and then follow the list. This specific protocol allows for easy handling with information about the amount, the purpose, the tube, and the target institute for analysis

If an acute infection is suspected, cell count, macroscopic assessment, and microscopy after gram staining help to make a quick diagnosis (within hours) and support a quick decision-making process for further treatment (Fig. 1).

The interpretation of the joint fluid sample can be done according to Trampuz et al. [74] and Stutz et al. [73], who proposed the following criteria: the main distinctive feature between reactive and septic arthritis is the number of cells. If this is greater than 20,000/µl, there is a high probability of an infectious event (Table 3). However, there are some limitations to these criteria. The cell count must be interpreted with regard to the individual patient, i.e., a leukocyte count of 15,000/µl can already be considered critical if an intraarticular implant is present (anchor or suture material) and the cut-off value due to presence of joint replacements is even more strict (physiological < 2800 leukocytes/µl). Additionally, in patients with immunosuppression, the leucocyte count may not be elevated and therefore mask a joint infection.

This interpretation is also supported by the systematic review of Margaretten et al. [48] who showed that a progressively higher synovial white blood cell (WBC) count increased the likelihood of septic arthritis (Table 4). Additionally, they could show the importance of polymorphonuclear cells with an increased likelihood for septic arthritis when the percentage of polymorphonuclear cells is at least 90% (LR 3.4; 95% CI 2.8–4.2) [48]. If the polymorphonuclear cells are less than 90%, the likelihood decreased (LR 0.34; 95% CI 0.25–0.47) [48].

**Table 4:** Likelihood ratio of septic arthritis according to the synovial white blood cell count (LR = likelihood ratio, CI = confidence interval) [48]

Synovial WBC count	
< 25.000 μL	LR 0.32; 95% CI 0.23–0.43
> 25.000 μL	LR 2.90; 95% CI 2.5–3.4
> 50 000/μL	LR 7.70; 95% CI 5.7–11.0
> 100 000/μL	LR 28.0; 95% CI 12.0–66.0

The negative results after cultivation, for assessment of joint fluid pathogens in the sample, do not necessarily exclude an infection. This also applies to the long-term cultivation (14 days and longer) [35].

### Further microbiological diagnostics

In addition to the initially obtained joint fluid sample, revision arthroscopies should also collect at least 5 (tissue) samples for further microbiological investigation. The sensitivity for a germ detection is significantly increased with tissue samples compared to joint fluid only [87]. It should also be noted that bacterial detection is significantly less frequent with an ongoing antibiotic therapy. Therefore, if a joint infection is suspected, the main aim is to check for pathogens before starting an empirical i.v. antibiotic therapy. If the situation requires an implant removal during the revision surgery, it is recommended to prepare the implant(s) for an additional microbiological assessment using sonication. The sensitivity and specificity of sonication exceeds that of tissue biopsies (79% versus 61% for tissue biopsy) with a high specificity of 99% in total joint explants [59]

Additionally, positive microbiological results should also be interpreted with regards to a possible false-positive result and be discussed with the microbiologist and infectious disease specialist.

In any case, a long-term culture (at least 14 days) of the samples is recommended, as some pathogens can only be detected after this time period of cultivation. Specifically, *Cutibacterium acnes* is frequently detected in shoulder joint infections [49]. In state-of-the-art microbiological institutes, 16S ribosomal RNA PCR (polymerase chain reaction) can be used as a reliable (high sensitivity) and fast diagnostic tool that allows the detection of a broad range of pathogens with pathogen-specific PCR [46].

## Classification of septic arthritis

**Fig. 2:**
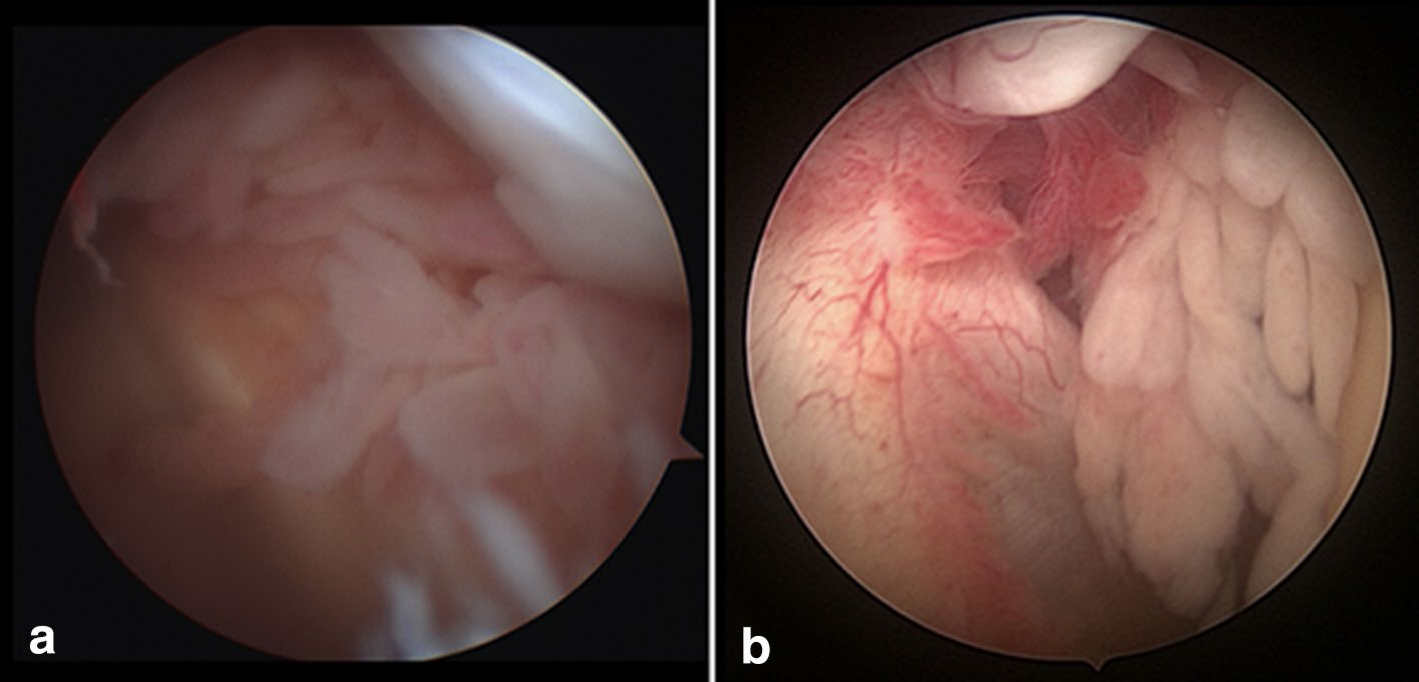
**a** Early detected knee joint infection after arthroscopy (Gächter type I) with clear synovialitis, and **b** shoulder joint infection after arthroscopic irrigation, before debridement (Gächter type II) with clear synovialitis and petechial bleeding in the anterior joint compartment with fibrin deposits

Several classifications are available which evaluate the joint infection according to pathological, anatomical [25], clinical [73], or arthroscopic [30] aspects. The most frequently used classification with clinical relevance is the classification according to Gächter (Table 5, Fig. 2).

**Table 5:** Classification of a joint infection according to Gächter [30]

I. Cloudy effusion, synovialitis, and possible petechial bleeding—no visible changes on radiographs
II. Clear synovialitis, putrid effusion, and fibrin deposits (Fig. 1a, b)—no visible changes on radiographs
III. Villi formation ("bath sponge") and chambering—beginning of cartilage damage with no visible changes on radiographs
IV. Aggressive synovial infiltration with undermining of the cartilage—radiological: osteolysis and cysts

### Therapeutic approach

If an infection is confirmed or suspected, an early arthroscopic joint irrigation and joint debridement should be performed (Fig. 2). The patient should be operated on within a few hours, if the patient has intervention-related fever and/or an increased cell-count analysis after joint puncture.

**Fig. 3:**
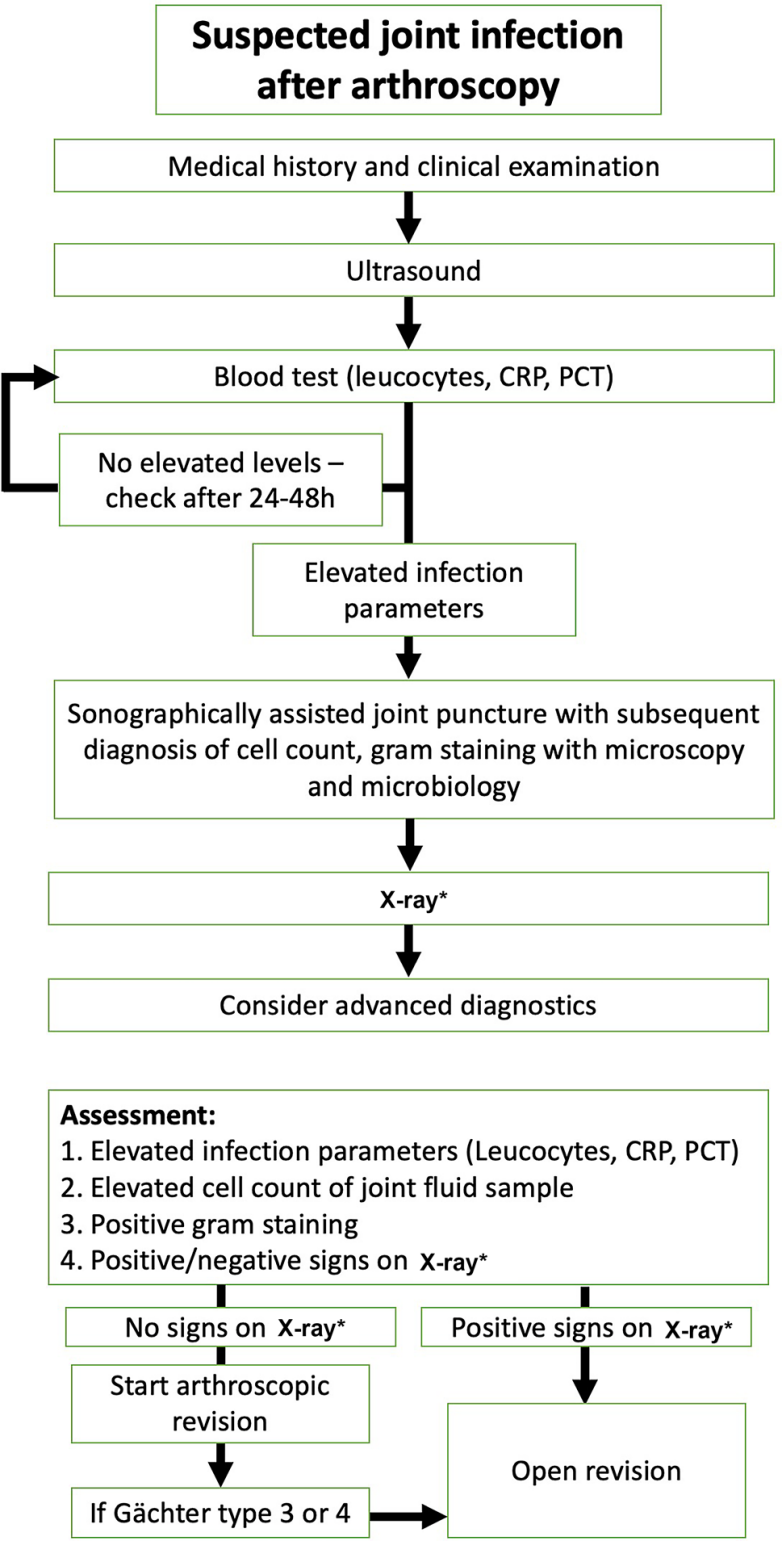
Algorithm for suspected joint infection after arthroscopy. In cases with indwelling implants, it is important to distinguish between an acute and chronic infection (see Table 4) in regard to implant preservation or removal (* in chronic cases mandatory, in acute cases helpful to identify implants and their position in case of surgery and subsequent removal if patient is not known to the presenting surgeon

If a high-grade joint infection is already confirmed at the time of diagnosis (Gächter stage 4) by osteolysis using conventional radiography, an open procedure should be considered (Fig. 3) [27].

At least five tissue samples should be obtained intraoperatively before starting a calculated antibiotic therapy. In addition, the histological examination is essential to support the diagnosis and to differentiate between septic arthritis between non-infectious joint pathologies (e.g., gout arthropathy) [39].

### Surgical therapy

During revision, extensive lavage, debridement with synovectomy and hemostasis should be performed. Necrotic tissue or pannus tissue should be carefully removed.

In acute infections that are described in most arthroscopic case, an implant-retaining strategy with irrigation, debridement, and synovectomy followed by anti-biofilm antibiotic treatment should be targeted. In the case of chronic infections, complete hardware removal is necessary in most cases.

The surgical strategy aims to proceed according to the stage of the infection (Gächter I–IV). In the further post-operative course, a "second look" may be necessary. This mainly depends upon clinical signs and laboratory parameters. The intra-articular drain can give information about the joint fluid (clear or cloudy) and the infection blood parameters should drop after surgery (CRP, leucocytes, PCT) during antibiotic administration.

In the case of severe infection with residual infection parameters, a second surgery is required. It cannot be confirmed whether the repeated biopsy during the “second look” is clinically meaningful. Therefore, no recommendation can be made, as an ongoing antibiotic therapy will have a major impact on the microbiological results.

The intraoperative lavage should be carried out with a sufficient fluid volume (6 L of NaCl recommended). Antiseptics such as iodine-containing solutions, chlorhexidine, or hydrogen peroxide have good antimicrobial effects, but must not be used during surgical joint intervention due to their high chondrotoxicity that could lead to advanced chondrolysis [60, 63].

Drainage (with suction) is recommended to control the remaining intraarticular fluid and to have a direct visualization of the fluid itself, which may help to evaluate the post-operative clinical course [41]. The application of a suction–irrigation drainage or the application of a vacuum dressing is not recommended for intra-articular infections.

### Antibiotic therapy

The administration of intra-articular antibiotics is not recommended, since the local effect level with systemic administration is above the minimum inhibitory concentration [52]. Additionally, there may also be an increased chondrotoxicity when administered locally.

Following adequate tissue and joint fluid collection, a calculated systemic antibiotic therapy must be started intravenously. In the absence of other risk factors, a second-generation cephalosporin is recommended for an antibiotic therapy of joint infections. However, newer strategies suggest the expansion of the calculated antibiotic therapy and the "hit hard and early" strategy. This will include the i.v. application of piperacillin/tazobactam (3 g) or amoxicillin/clavulanic acid (2.2 g) three times a day [31, 77]. Particularly in cases of acute infections with the intention to preserve implants, a biofilm effective antibiotic, such as rifampicin (dry wounds), in combination with the calculated antibiosis is recommended [86].

After receiving the antibiogram, the specific antibiotic therapy should be performed. The choice of antibiotic, as well as the method of application (i.v. vs. p.o.) and duration of the therapy, always depend upon accompanying factors. These can be the duration and severity of the infection, as well as accompanying diseases of the patient [31]. Special therapy regimes must be implemented when detecting multi-resistant bacteria and a special attention is required for rifampicin and ciprofloxacin resistant bacteria, due to their importance in treating biofilms. Therefore, an interdisciplinary cooperation between multiple faculties is recommended to find the best treatment for the patient.

### Aftercare

During the duration of post-operative care, the passive mobilization of the joint is of high importance and joints should not be immobilized [43]. After removing the drainage and the recovery of the infection parameters, a more passive-assistive therapy can be started. With further control of the infections and improvement of joint conditions, active mobilization can be started. The further rehabilitation treatment is then based on the intraoperative findings and the reconstructive procedures during surgery.

## Conclusions

In conclusion, septic arthritis is a significant complication after arthroscopic surgery. A major challenge in diagnostics is the differentiation of the post-operative status between a non-infected hyperinflammatory joint versus septic arthritis. Therefore, joint puncture for microbiological evaluation and particularly for fast leukocyte cell-count diagnostics is advocated. A cell count of more than 2.000 leukocyte/µl with more than 70% of polymorphonuclear cells is the generally accepted threshold for septic arthritis. The therapy is based on an arthroscopic or open surgical approach in combination with an adequate antibiotic therapy for 6–12 weeks.
